# Causes and circumstances of accidents at work in the European Union, Slovakia and Czech Republic

**DOI:** 10.3389/fpubh.2023.1118330

**Published:** 2023-06-20

**Authors:** Katarína Hollá, Alena Ďad'ová, Mária Hudáková, Jirí Valla, Anna Cidlinová, Linda Makovická Osvaldová

**Affiliations:** ^1^Faculty of Security Engineering, University of Zilina, Zilina, Slovakia; ^2^Occupational Safety Research Institute, Prague, Czechia

**Keywords:** occupational safety and health, occupational accidents, Slovakia, Czech Republic, European Union, risk assessment, survey

## Abstract

There are several challenges in occupational safety and health that need to be addressed. The basic premise is the reduction of occupational accidents in individual sectors. Finding effective tools to reduce them is very challenging. Safety culture is perceived differently in the countries of the European Union. The basic intention of this article is to compare the accidents number in these two countries and in the European Union in selected NACE categories. This comparison is based on the statistical processing of data by NACE category and representation of accident rates in individual industries. The main causes of accidents were identified, which give space for further research in this field a state measures to prevent work accidents to happen or to reduce its numbers.

## 1. Introduction

The Slovak Republic (SR) and the Czech Republic (CR) are part of the European Union (EU), in the past they formed one state until 1992. Nowadays, the number of inhabitants in the Slovak Republic is around 5.459 million inhabitants and in the Czech Republic 10.7 million inhabitants. Based on this, it can be concluded that the Czech Republic is twice as large as the Slovak Republic. Both countries joined the European Union in 2004. The purpose of this article is to compare how the countries have developed in the area of occupational safety and health (OSH), with an emphasis on comparing the number of occupational accidents for certain period and identifying causes of their occurrence. In the context of the European Union, the concept of OSH has a relatively broad definition. This definition OSH in the European Union includes good working conditions for the employee, prevention of diseases and prevention of accidents at work. In short, health and safety promotes “the adaptation of work to the person and of each person to their job” (1). OSH in the SR is characterized as a state of the workplace where a certain possibility of threatening the health or life of persons, destroying or damaging economic values will be excluded or reduced under the conditions of compliance with the rules, whether safety requirements or technological work procedures, which are valid for the respective work process and workplace and without the effects of unpredictable external influences (2, 3). OSH in the CR is a field that deals with technical, technological, organizational, educational and other measures, the aim of which is to create such a workplace, working environment and work in which occupational accidents will not occur. Work safety or safety at work is the state of working conditions preventing the impact of dangerous factors of the work process on employees, or other persons. In conclusion whatever the source of the definition of this term, it is always focused on employees, its basic goal is the prevention of occupational accidents and it refers to the work process and the workplace (4, 5).

Competent authorities must have control over crucial OSH issues, both at the international and national level. Table 1 shows the European and national scope of competent authorities in the field of OSH.

**Table 1:** Management model and institutions responsible for OSH in the EU, SR and the CR.

	**Legislative power**	**Executive power**	**Decentralization of OSH management (local level)**
European Union	European Union information agency for occupational safety and health (EU—OSHA)	EU-OSHA Administrative Board and Executive Board EU Member States	EU member states
Slovak Republic	Ministry of Labor, Social Affairs and Family—occupational safety Ministry of Health—health protection	Work inspection National Labor Inspectorate—manages and directs labor inspection Public Health Authority	Labor inspectorates in the regions Regional Public Health Office
Czech Republic	Ministry of Labor and Social Affairs—peace of mind Ministry of Health—health protection	State Labor Inspection Office Ministry of Health (chief hygienist)	Regional labor inspectorates (OIP) Regional hygiene stations

European directives set minimum standards for safety and health protection in the workplace. EU directives are implemented through the national legislation of the member states. Member States may adopt stricter regulations to protect workers, but their legislation must meet minimum standards. For this reason, national safety and health legislation varies within the EU. Table 2 shows selected basic legal regulations and standards established by the EU and their implementation into the legal environment of the Czech Republic and Slovakia.

**Table 2:** Legal regulations and standards in the area of health and safety at work.

	**Legislation**	**Regulation/decree**	**Technical standard (management)**
European Union	Framework Directive 89/391/EEC	Council Directive 89/654/EEC of 30 November 1989 concerning the minimum safety and health requirements for the workplace	ISO 45001:2018 Occupational health and safety management systems—requirement with guidance for use
Slovak Republic	Act 124/2006 Coll. on safety and health protection at work	Regulation of the Government of the Slovak Republic No. 391/2006 Coll. on minimum safety and health requirements for the workplace STN	ISO 45001: 2019 Safety and security management systems health at work
Czech Republic	Law no. 309/2006 Coll., on ensuring other conditions of safety and health protection at work Government	Regulation no. 361/2007 Coll. Government Regulation establishing the conditions of health protection at work	CSN ISO 45001 (010801) Safety and health management systems at work

The OSH is also captured in the Constitutions of the member states and is further established by legal regulations, internal and other regulations for ensuring safety and health protection at work. The basic ones are listed in Table 2. There are many more, but for the purposes of this article it is important to list them.

## 2. Materials and methods

As mentioned, SR and the CR joined the EU in the same period. In the following text, we will compare their accident rate from the point of view of economic activities according to NACE. NACE stands for Classification of Economic Activities. The CZ-NACE and SK-NACE classification was developed according to the international statistical classification of economic activities, in accordance with the Regulation of the European Parliament and the Council (EC) No. 1893/2006 of December 20, 2006, which introduces the statistical classification of economic activities NACE (6).

As part of the statistical evaluation, we used the databases of the European Union, the Slovak Republic and the Czech Republic in 9 years' period:

- Analysis of occupational accidents for Czech Republic.
- Analysis of occupational accidents for European union.
- Analysis of occupational accidents for Slovak Republic.

OSH is built on the principle of prevention, i.e., the basic duty is to act in such a way that there are no occupational accidents or occupational diseases. An accident at work is defined in ESAW methodology as a discrete occurrence during the course of work which leads to physical or mental harm. Fatal accidents at work are those that lead to the death of the victim within one year of the accident taking place. Non-fatal accidents at work are defined as those that result at least four full calendar days of absence from work (they are sometimes also called “serious accidents at work”). Non-fatal accidents at work may result in a considerable number of working days being lost and often involve considerable harm for the workers concerned and their families. They have the potential to force people, for example, to live with a permanent disability, to leave the labor market, or to change job (7). The same is for the Slovak Republic and The Czech Republic.

In the next part, we evaluated the most dangerous industries from the point of view of accidents.

The causes of injuries are then directly linked to the statistics we present and are an important part of developing measures to prevent injuries. We have obtained this information from:

- European Survey of Enterprises on New and Emerging Risks (ESENER 1, 2 and 3) (https://osha.europa.eu/en/facts-and-figures/esener).
- Slovak Survey (Risk Assessment in OSH) ran in 2023 (in accordance with the international quality standards of WAPOR, ESOMAR and the standards of the Slovak Association of Research Agencies).
- Czech Survey (8).

**Figure 1:**
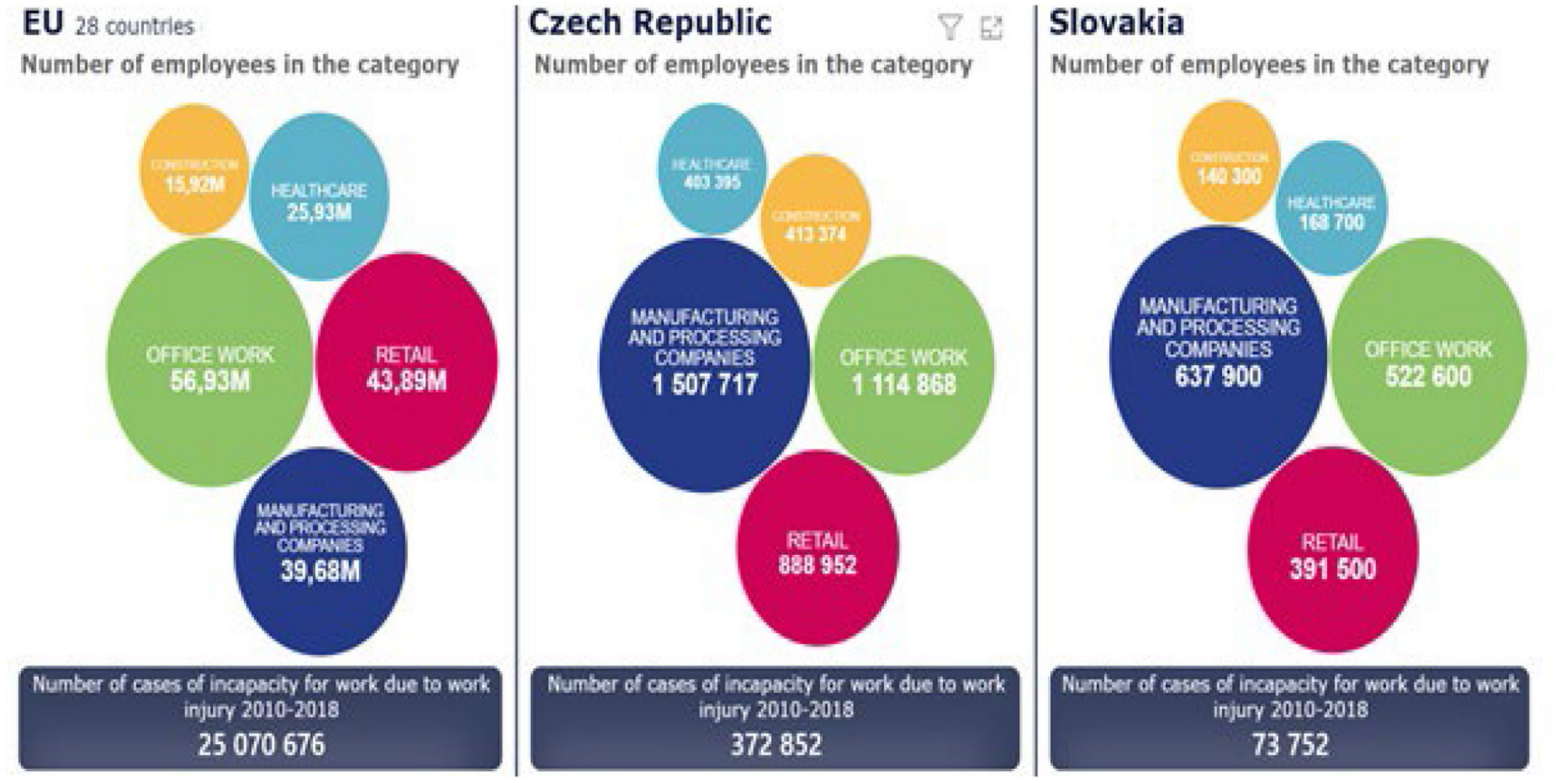
Number of employees in selected categories and the total number of registered occupational accidents in the EU, Slovakia and the Czech Republic 2010–2018 (9–12).

**Figure 2:**
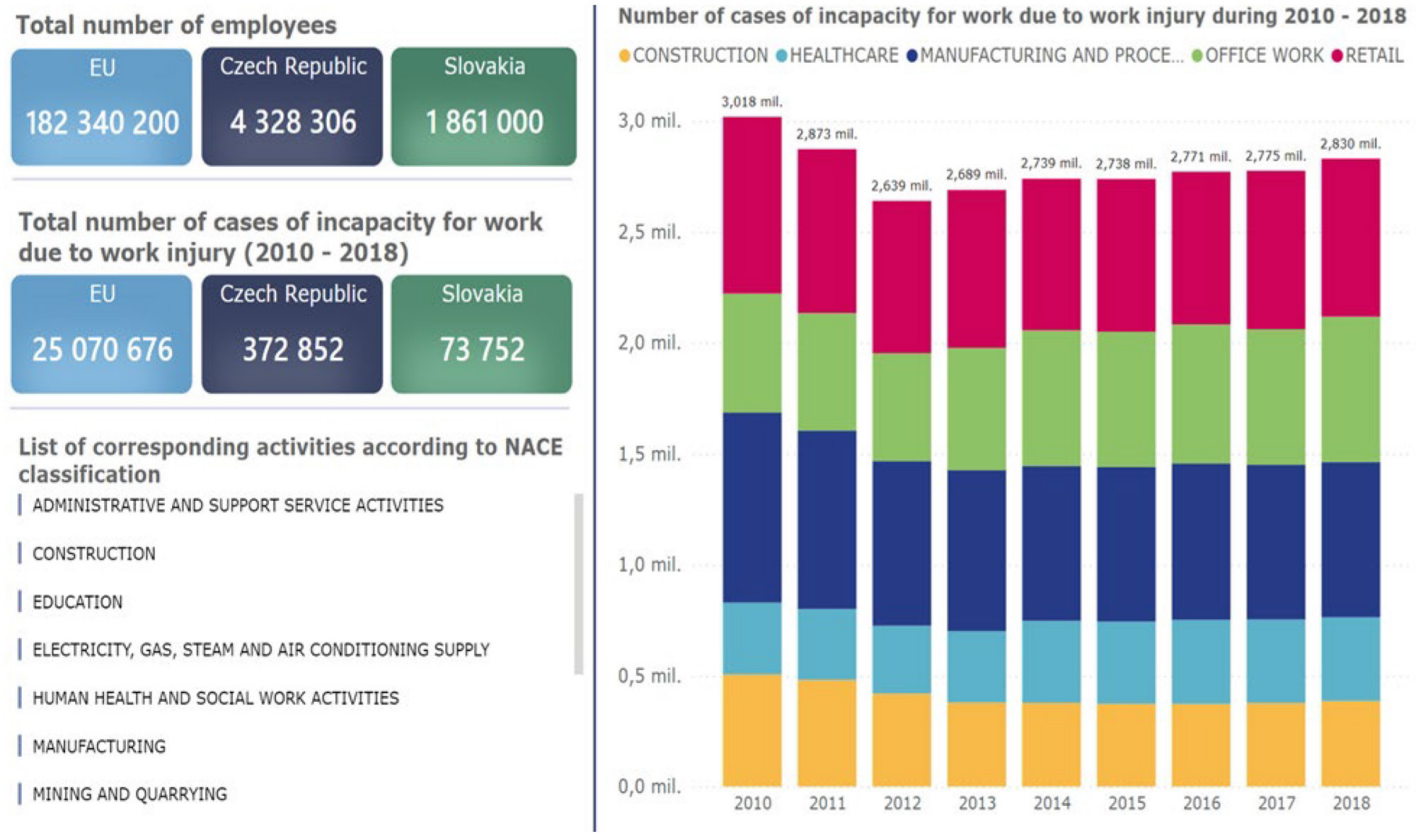
Total number of employees in the EU, the Czech Republic and the Slovak Republic and the number of registered occupational accidents in selected categories in the period 2010–2018 in EU (9–12).

We also used Microsoft Power BI: Data Visualization for showing the results in Figures 1, 2. This tool is good for the data visualization and software will generate a variety of different charts, graphs, and map types via a simple, intuitive dashboard interface. Largest number of workers in categories NACE (Table 3).

**Table 3:** Assessment of the largest number of workers in categories NACE in EU, SR and CZ (2010–2018).

	**Largest number of workers in categories NACE**
European Union	1. Office work
	2. Retail
	3. Manufacturing and processing companies
	4. Healthcare
	5. Constructions
Slovak Republic	1. Manufacturing and processing companies
	2. Office work
	3. Retail
	4. Healthcare
	5. Constructions
Czech Republic	1. Manufacturing and processing companies
	2. Office work
	3. Retail
	4. Constructions
	5. Healthcare

## 3. Results

In the following text, we will assess the number of employees in the EU, the SR and the Czech Republic and give the total number of accidents for the years 2010–2018 (Figure 1). For this period, common data were available for the establishment of a comparison file. In Figure 1 we present data of the European Union, the Czech Republic and the Slovak Republic from the point of view of the representation of the number of workers in individual industries according to NACE.

In Slovakia and the Czech Republic, the first place from work accidents occurrence belongs to Manufacturing and processing companies. Office work is in first place in the European Union.

The number of registered occupational accidents in the EU, the Czech Republic and the Slovak Republic is not low (Figure 2). From the point of view of the given years in Slovakia, the statistics for the given years have not changed significantly, the difference between the lowest number of occupational accidents in 2014 and the highest number of registered occupational accidents in 2018 in the monitored period is 1,649 registered occupational accidents. Within the European Union, registered occupational accidents have been increasing since 2012. In the Czech Republic, the highest number of accidents was registered in the years 2015–2018. The given statistics include all enterprises in terms of size.

## 4. Discussion

For comparison with previous assessment, we present the main conclusions from the ESENER surveys. EU-OSHA's European Enterprise Survey on New and Emerging Risks (ESENER) is a large-scale survey of how health and safety risks are managed in European workplaces. In conclusion, we want to follow up on the issue of the statistics of occupational accidents, the connection to the causes of their occurrence and the main problems.

Three ESENER surveys have been carried out in the European Union, led by the Agency for Safety and Health at Work (13). On the basis of surveys, it was determined how European workplaces manage risks and how they manage the field of safety and health protection at work. Thousands of companies and organizations in the European Union were involved in the surveys. Organizations and businesses were presented with a questionnaire that focused on the following topics: the impact of general risks on the field of OSH and the possibility of their management, psychosocial risks and other factors such as stress, harassment and bullying, and employee participation in OSH procedures (14).

Surveys have found that new technologies, social conditions, economic changes, an aging workforce, home office, language comprehension pose a particular problem in the workplace.

The ESENER 1 survey in 2009 shows that companies do not carry out risk assessments because they are unnecessary or due to a lack of expertise. According to a survey from 2009, the main concerns in the field of health and safety in companies are: diseases and damage to the musculoskeletal system, accidents, stress. From the ESENER 2 survey in 2014, psychosocial risks are considered to be the most risk factors. The survey found that almost one in five organizations have a problem with psychosocial risks and do not have sufficient information or appropriate tools to eliminate them. As part of the risk assessment in the ESENER 2 survey, several pieces of information were found. According to the results of the survey, 76% of EU-28 organizations regularly perform risk assessment. Up to 90% of the surveyed organizations say that risk assessment is a useful way of managing safety and health at work. As part of the ESENER 3 survey, they were identified as the most affecting problems related to the MSDs (14–17).

Statistics show that most fatal occupational accidents occur in small and micro-enterprises (14–17).

Main 5 causes of the work accident 2010–2018 in Czech republic are as follow:

1. Poorly or inadequately assessed risk
2. Use of unsafe practices in the workplace
3. Defective or adverse condition of the source of injury
4. Failure to use or inconsistent use of personal protective equipment
5. Threats from others (distraction at work, joking, arguing).

Main 5 causes of the work accident 2010–2018 in Slovak Republic are as follow:

1. Shortcomings in the personal requirements for proper job performance
2. Defective or adverse condition of the source of injury
3. Use of unsafe practices in the workplace
4. Threats from others (distraction at work, joking, arguing)
5. Threats from animals and natural elements.

**Figure 3:**
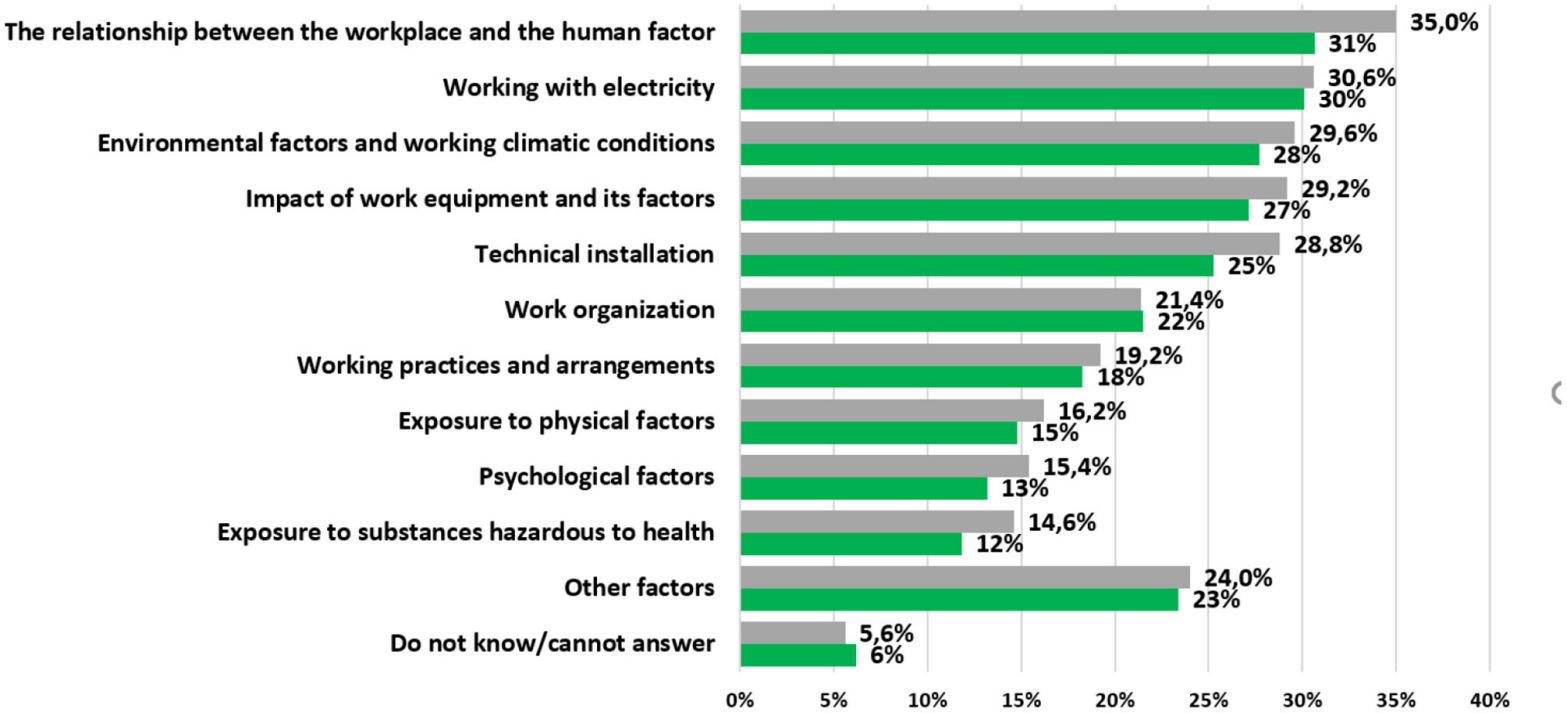
The most frequent safety risks perceived by micro enterprise (green) vs. survey sample.

We (University of Zilina) have just completed a survey of OSH businesses and have found out from around 500 micro and small businesses how they perceive the causes of work accidents in the Slovak Republic. This was a multiple choice (max 5) so the total exceeds 100%. The results are shown in Figure 3.

Figure 3 shows that the most frequent occupational accidents in micro enterprises are caused by the relationship between the workplace and the human factor.

**Figure 4:**
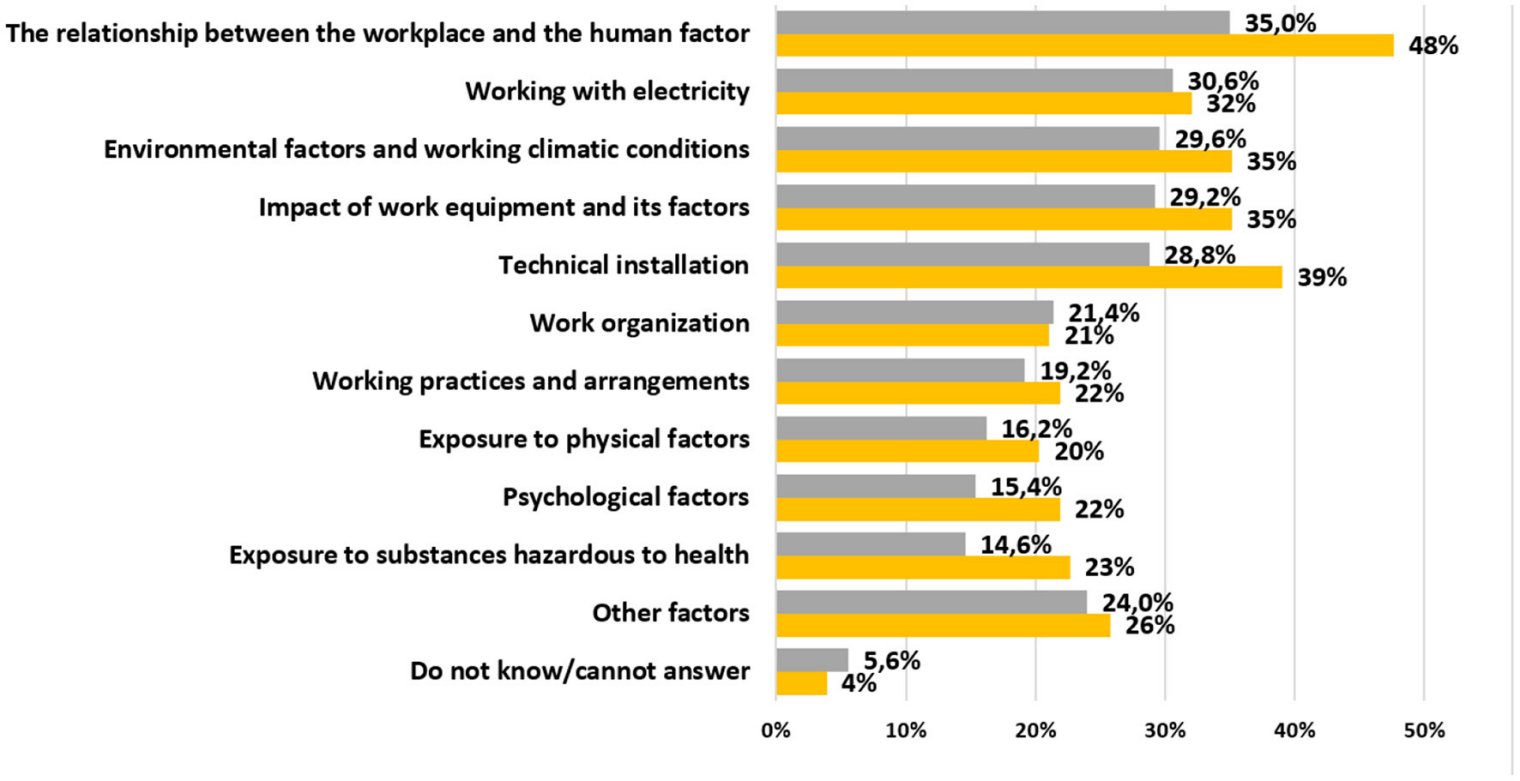
The most frequent safety risks perceived by small enterprise (orange) vs. survey sample.

Figure 4 shows that the most frequent occupational accidents in small enterprises are caused by the relationship between the workplace and the human factor.

What was quite surprising for us was that psychological factors were at the end of the list in both graphs, taking into account that after the COVID – 19 this belong into the most challenging issues in companies.

## 5. Conclusion

Based on the analysis of the causes of accidents, the main causes of occupational accidents in the EU, the Czech Republic and the Slovak Republic were determined in the years 2010–2018:

- Insufficient Risk Assessment,
- Lack of personal prerequisites for proper work performance,
- Using unsafe work practices or work methods.

Occupational accidents occur and will occur in case of insufficient prevention and preventive measures to reduce risks. By statistical comparison, we can identify problem areas that need to be focused on. European countries process such statistics according to European NACE industries, and therefore these data can be compared. In the article, Office work was identified as the main problem area for the European Union, and Manufacturing and processing companies in Slovakia and the Czech Republic. Insufficient Risk Assessment, lack of personal prerequisites for proper work performance and using unsafe work practices or work methods can be identified as the main causes. ESNER surveys indicate that companies are aware of the need for active work in the field of OSH, but they lack the appropriate tools to solve these problems. One of the more vulnerable groups are micro-enterprises and small businesses. In those large and medium-sized enterprises, it is almost necessary nowadays to introduce a Safety culture, and therefore we see as one of the possible solutions to apply this philosophy, at least in part, precisely in small and micro-enterprises.

Safety culture refers to the interaction between the requirements of the Safety Management System (SMS), how people make sense of them, based on their attitudes, values and beliefs, and what they actually do, as seen in decisions and behaviors. An organization's culture can have as big an influence on safety outcomes as the safety management system. “Safety culture” is a subset of the overall company culture (and is defined in the box on the right). Many companies talk about “safety culture” when referring to the inclination of their employees to comply with rules or act safety or unsafely (18, 19). However, we find that the culture and style of management is even more significant, for example a natural, unconscious bias for production over safety, or a tendency to focussing on the short-term and being highly reactive.

## Data availability statement

The original contributions presented in the study are included in the article/supplementary material, further inquiries can be directed to the corresponding author.

## Author contributions

KH: article structure, statistical assessment and analysis, comparison of selected indicators of the structure of employees and occupational accident rate in the European Union, and the Czech Republic and the Slovak Republic. AĎ: introduction preparing, comparison of selected indicators of the structure of employees and occupational accident rate in the European Union, and the Czech Republic and the Slovak Republic. MH: classification of enterprises according to NACE. LO: comparison of selected indicators of the structure of employees and occupational accident rate in the European Union and the Czech Republic and the Slovak Republic. AC: discussion, comparison of selected indicators of the structure of employees and occupational accident rate in the European Union, and the Czech Republic and the Slovak Republic. JV: comparison of selected indicators of the structure of employees and occupational accident rate in the European Union, the Czech Republic and the Slovak Republic, and conclusion. All authors contributed to the article and approved the submitted version.
